# The Use of Oil to Still the Waves

**Published:** 1889-04

**Authors:** 


### THE USE OF OIL TO STILL THE WAVES.

Under the above title Lieutenant W. H. Beehler contributes to the March CENTURY an account of the experiments made with oil in rough water. We quote the following account of two rescues: "The captain of the ship *Martha Cobb*, loaded with petroleum, fell in with a sinking vessel during a heavy gale in the North Atlantic in December 1886. The signal made stated the vessel was sinking and that all her boats had been stove. The *Martha Cobb* had lost her large boats, her bulwarks washed out, and decks swept in the same storm; the only boat left was a small sixteen foot dingey, which could not possibly live in the sea that was then running. The captain says he was puzzled and lay by for some hours hoping that the gale would moderate; but as there was no appearance of better weather and night coming on he decided to make an attempt to rescue the crew of the sinking vessel. The *Martha Cobb* had a cargo of petroleum, some of which leaked, and the captain had noticed that the sea in the wake of the ship was much smoother when the pumps were worked.

"He signaled to the other vessel to haul by the wind while he luffed to get to windward, and at the same time started the pumps; but the ship drifted faster than the oil, and while the oil made the sea comparatively smooth to windward, it did not cover the sea to leeward. He then ran down across the other vessel's stern, hauled up close under her lee, and started the pumps again; at the same time also he emptied a five-gallon can of fish oil down the scuppers. The effect was magical. In twenty minutes the sea between and around the vessels were broken down. The long heavy swell remained, but the combers and breaking seas were all gone. The little dingey with three men had no difficulty in pulling to windward, and the crew were saved. The boat was deeply loaded and did not ship any water, although the sea was breaking fiercely outside of the 'charmed' space in which the vessels lay on oiled seas.

"In June, 1885, the British ship *Slivemore* took fire and had to be abandoned when eight hundred miles north-east of the Seychelle Islands, Indian Ocean. The people took to the boats and made for Seychelle Islands. The third day after leaving the vessel a cyclone came up, and no one believed that the boats would live through it. Before they left the ship the boats had been supplied with oil for just such an emergency. Each boat got out a drag made of spars and oars lashed together, for what is known as a sea-anchor. Oakum saturated with petroleum was stuffed in long stockings hung over the bows of the boats. Before the oil was used the boats had been several times nearly filled with water and the occupants had to bail for their lives; but when oil was applied no further trouble was experienced. An oil-slick formed around the boats, which rode in perfect safety on tremendous swells which took the place of the previously breaking seas. Little if any water came over the sides of the boats, and the occupants could lie down and sleep. The boats eventually reached the islands, but every soul would have perished except for the forethought of Captain Comby, the captain of the *Slivemore*."

